# Prognostic Value of [18F]-Fluoro-Deoxy-Glucose PET/CT, S100 or MIA for Assessment of Cancer-Associated Mortality in Patients with High Risk Melanoma

**DOI:** 10.1371/journal.pone.0024632

**Published:** 2011-09-14

**Authors:** Markus Essler, Anna Link, Benedetta Belloni, Vesna Mirceva, Michael Souvatzoglou, Markus Thaler, Bernhard Haller, Ruediger Hein, Bernd J. Krause

**Affiliations:** 1 Nuklearmedizinische Klinik und Poliklinik, Klinikum rechts-der-Isar, Technische Universität München, München, Germany; 2 Dermatologische Klinik und Poliklinik, Technische Universität München, München, Germany; 3 Institut für Medizinische Statistik und Epidmiologie, Technische Universität München, München, Germany; 4 Institut für Klinische Chemie und Pathobiochemie, Technische Universität München, München, Germany; University of Texas, M.D. Anderson Cancer Center, United States of America

## Abstract

**Purpose:**

To assess the prognostic value of FDG PET/CT compared to the tumor markers S100B and melanoma inhibitory activity (MIA) in patients with high risk melanoma.

**Methods:**

Retrospective study in 125 consecutive patients with high risk melanoma that underwent FDG PET/CT for re-staging. Diagnostic accuracy and prognostic value was determined for FDG PET/CT as well as for S100B and MIA. As standard of reference, cytological, histological, PET/CT or MRI follow-up findings as well as clinical follow-up were used.

**Results:**

Of 125 patients, FDG PET/CT was positive in 62 patients. 37 (29.6%) patients had elevated S100B (>100 pg/ml) and 24 (20.2%) had elevated MIA (>10 pg/ml) values. Overall specificities for FDG PET/CT, S100B and MIA were 96.8% (95% CI, 89.1% to 99.1%), 85.7% (75.0% to 92.3%), and 95.2% (86.9% to 98.4%), corresponding sensitivities were 96.8% (89.0% to 99.1%), 45.2% (33.4% to 55.5%), and 36.1% (25.2% to 48.6%), respectively. The negative predictive values (NPV) for PET/CT, S100B, and MIA were 96.8% (89.1% to 99.1%), 61.4% (50.9% to 70.9%), and 60.6% (50.8% to 69.7%). The positive predictive values (PPV) were 96.7% (89.0% to 99.1%), 75.7% (59.9% to 86.6%), and 88.0% (70.0% to 95.8%). Patients with elevated S100B- or MIA values or PET/CT positive findings showed a significantly (p<0.001 each, univariate Cox regression models) higher risk of melanoma associated death which was increased 4.2-, 6.5- or 17.2-fold, respectively.

**Conclusion:**

PET/CT has a higher prognostic power in the assessment of cancer-associated mortality in melanoma patients compared with S100 and MIA.

## Introduction

Accurate staging and follow-up in melanoma patients is essential for appropriate treatment planning and may improve survival [1], [2], [3]. Several parameters with potentially prognostic value have been identified in melanoma. These include Breslow thickness, ulceration histological subtype and tumor status of regional lymph nodes [4]. High risk melanoma patients have a recurrence and mortality risk higher than 35% to 50% within five years [5], [6].

Serum S100B is a valuable prognostic marker for assessment of melanoma mortality [7], [8]. More than 80% of patients with metastatic melanoma have elevated S100B levels. Rising levels of S100B are a useful predictor of a relapse of disease and were found to give readings 5–23 weeks before other clinical or conventional radiological methods. Melanoma inhibitory activity (MIA) is a marker for progression from localized to metastatic disease in advanced melanomas. MIA possesses a higher specificity in the detection of metastatic disease compared to S100B and is negatively correlated with survival [7], [8]. The serum lactate dehydrogenase (LDH) level is the currently most important serum marker of poor prognosis in melanoma patients and has been recently included into the AJCC staging system [2].

[^18^F]-fluoro-deoxy-glucose positron emission tomography (FDG-PET) is successfully used for primary staging of patients with malignant melanoma [9], [10]. FDG-PET/CT has a high accuracy for staging in high risk melanoma patients and is superior to conventional imaging methods [11], [12], [13]. Sensitivity and specificity of PET/CT in staging of malignant melanoma are approximately 98%. However, its role in follow-up is controversial and it is important to identify clinical situations where PET/CT has a therapeutic impact. In patients with high risk melanoma PET/CT may help to detect recurrence or metastatic disease and to initiate a specific therapy. The prognostic value of PET/CT in high risk melanoma patients compared to the tumor markers S100B and MIA has not been assessed [14].

## Methods

### Ethics statement

The retrospective study was approved by the “Ethical Board” of the “Technische Universität München”. Written informed consent was available from all patients.

### Participants

125 consecutive patients with suspected metastases of malignant melanoma were included between November 2003 and May 2006. Patients who had a Breslow tumor thickness >2.0 mm, elevated S100B or MIA level, a sentinel lymph node positive for tumor or a known/resected metastasis in the history were selected. All patients were AJCC stage IIB or higher or had suspicious findings in ultrasound. The age of the patients was in mean 58.8±14.1 years, 58 were female (46.4%) and 67 (53.6%) patients were male (Table 1).

**Table 1:** Patient characteristics.

Sex	Age	T-stadium	Metastasis
male: 58 (56.4%)	mean age: 58.8 years	T1: 24	Skin: 7
female: 67 (53.6%)	range: 26.4–88.	T2: 26	Lymph nodes: 33
		T3: 26	visceral: 22
		T4: 26	no metastasis: 63
		CUP: 23	

### PET/CT-Imaging

Patients were scanned with a Biograph 16 PET/CT (Siemens Medical Solutions, Erlangen, Germany) after injection of [^18^F]-FDG (5 MBq/kg body weight). The scans were performed 60 minutes after injection of the tracer. Patients fasted six hours before the PET examination and the blood glucose level was <150 mg/dl in all patients. Whole body images were acquired from all patients, i.e. 15 bed positions á 2 minutes acquisition time. 59 patients received a contrast enhanced CT including the application of 300 ml Imeron as contrast media. Oral contrast media was administered 30 minutes before injection of FDG. Image analysis was performed using TrueD software (Siemens Medical Systems, Malvern, PA). All studies were analyzed independently by two experienced nuclear medicine specialists who were blinded to patient outcome. Prior clinical reports of these studies were not taken into account. In visual assessment focal, non physiological FDG-uptake was defined as consistent with tumour tissue. For quantitative analysis of FDG-uptake standardized uptake values (SUV) were measured using 3 dimensional regions of interest in TrueD software.

### Standard of Reference

Lymph node or distant metastases found by PET/CT were confirmed by histology, by follow up PET/CT, MRI, by clinical follow for up to 18–48 month or tumor associated death. A false positive PET/CT diagnosis was determined if histology of the lesion or clinical follow-up ruled out metastases (disappearance of lesions without therapy) or if MRI indicated a different etiology. False negative metastases were confirmed by clinical follow-up, death of the patient or MRI.

### Measurement of the tumor markers S100 and MIA

S100B-levels were determined with a commercial sandwich electrochemiluminescence immunoassay (ECLIA) of the Elecsys 2010-system from Roche (Mannheim, Germany). With an interassay coefficient variation of 2.0 to 2.8% in human serum (manufacturer's information) the measuring range reaches from 5 to 3900 pg/ml. As recommended by the manufacturer, samples with S100B-concentrations >3900 pg/ml were diluted 1 ∶ 5 with the low calibrator of CalSet S100 (Roche). For all measurements, a voluntary quality control according to the “Guidelines of the Federal Medical Association for quality assurance in laboratory medicine investigations” (RiliBÄK) was performed. The cut-off value for S100 was 100 pg/ml. The serum concentration of MIA was determined by ELISA using a commercial assay kit (Roche) according to the manufacturer's instructions. Briefly, two monoclonal antibodies directed against the NH2-terminal- (clone 2F7) conjugated with horseradish peroxidase or the COO- terminal-region (clone 1A12) conjugated with biotin were used. Ten microliters of serum or standard (recombinant MIA purified from Chinese Hamster Ovary cells, provided by the manufacturer) were incubated with 200 µl reagent containing both antibodies in streptavidin coated 96-well plates for 45 minutes with shaking. After washing three times with washing buffer (Roche) 200 µl of 2.2-azino-de(3-ethylbenz-thiazoline sulphonate (Roche) was incubated in the wells for 30 minutes and measured colorimetrically at 405 nm. Using the indicated standard concentrations between 0.1 ng/ml and 50 ng/ml. The 97 percentile was set as a cut-off at 10.0 ng/ml. Also LDH was measured by a commercial kit according to the manufacturer's instructions (Roche, Germany). The cut-off value was 244 U/l. The time between PET/CT and tumor marker measurement was in mean 16.7 days (median 9 days).

### Statistical analysis

Statistical analysis of data was performed using SPSS version 16. All tests were performed using an explorative two-tailed significance level of α = 0.05. For determination of sensitivity, specificity, positive and negative predictive values of S100, MIA, LDH or PET/CT, the diagnosis made by these modalities was compared to the standard of reference. 95% confidence intervals are presented for all relevant measures. To investigate the diagnostic accuracy of the tumor markers and to determine thresholds for identification of progressive disease Receiver Operating Characteristic (ROC) analyses were conducted. The Youden-Index ( = sensitivity + specificity − 1) was calculated to obtain optimal cut-off values. To determine the univariate correlation of S100B, MIA and LDH with survival quantitative values were dichotomized. Survival probabilities were estimated by Kaplan-Meier method. To evaluate the association of diagnostic modalities with survival and to estimate corresponding hazard ratios Cox regression models were fitted. The overall C index was determined as a measure of discrimination between events and non-events. The index represents the proportion of all usable pairs in which predictions and outcomes are concordant [15]. Values closer to one indicate better discrimination.

## Results

### Sensitivity and specificity of FDG PET/CT in the detection of recurrence/metastases of high risk melanoma

The standard of reference defined 62 patients (49.6%) as having recurrent or metastatic melanoma (Table 2). In FDG PET/CT, 62 patients were positive for tumor and 63 did not show metastases or recurrence. Two patients were false positive and two were false negative. The sensitivity and the specificity of FDG-PET/CT to detect tumor tissue in high risk melanoma patients was 96.8% each (95% confidence interval (CI) 89.0% to 99.1% and.89.1% to 99.1%) (Table 2).

**Table 2:** Patients positive or negative for recurrent/metastatasized melanoma in PET/CT, S100, MIA and standard of reference.

S100B	MIA	PET/CT	standard
elevated: 37	elevated: 25	recurrence:62	recurrence:64
not elevated: 88	not elevated: 99	no recurrence:63	no recurrence:61

### Sensitivity and specificity of the tumor markers S100B and MIA in the detection of recurrence/metastases of high risk melanoma

Out of 125 patients 98 had normal and 26 had elevated MIA levels. From one patient MIA was not available. 39 patients were false negative and 3 were false positive. Moreover, 88 patients had normal S100B and 37 had elevated S100B levels. 34 patients were false negative and 9 were false positive. In the group negative for tumour tissue according to the gold standard S100 was in mean 66.9±34.7 pg/ml (median 58 pg/ml, maximum 190, minimum 27 pg/ml). MIA was in mean 6.6±2.1 ng/ml (median 6.1 ng/ml, maximum 14.6, minimum 2.1 ng/ml). In the group positive for tumour tissue S100 was in mean 203±383 ng/ml (median 73 ng/ml, maximum 2285 ng/ml, minimum 23 ng/ml). The sensitivity and specificity for S100B were 45.2% (95% CI, 33.4% to 57.5%) and 85.7% (75.0% to 92.3%) and for MIA 36.1% (25.2% to 48.6%) and 95.2% (86.9% to 98.4%) (Table 3). In a subgroup of 58 patients LDH was also determined. Using a cut-off value of 244 U/l 33 patients had elevated LDH values and 25 had normal LDH values. The sensitivity to detect metastasis/recurrence was 30.3% (95% CI, 17.4% to 47.3%) and the specificity 96.0% (80.5% to 99.8%). A ROC-analysis revealed that the area-under-the-curve for S100, MIA and LDH were 0.685, 0.681 and 0.619.The NPVs of PET/CT, S100, and MIA were 96.8% (95% CI, 89.1% to 99.1%), 61.4% (50.9% to 70.9%), and 60.6% (50.8% to 69.7%). The PPVs were 96.8% (89.0% to 99.1%), 75.7% (59.9% to 86.6%), and 88.0% (70.0% to 95.8%). In a subgroup of 58 patients LDH was measured and a NPV of 51.1% (37.2% to 64.7%) and a PPV of 90.9% (62.2% to 99.5%) was found (Table 3). A multivariate analysis revealed that a combination of FDG PET/CT, S100B and MIA did not improve the sensitivity and specificity to detect metastases in high risk melanoma patients.

**Table 3:** Melanoma restaging.

	PET/CT	(95% CI)	S100B	(95% CI)	MIA	(95% CI)
**specificity**	96.8%	(89.0–99.1)	85.7%	(75.03–92.30)	95.2%	(86.91–98.37)
**sensitivity**	96.8%	(89.1–99.1)	45.2%	(33.42–57.47)	36.1%	(25.17–48.61)
**NPV**	96.8%	(89.1–99.1)	61.4%	(50.2–70.9)	60.6%	(50.8–69.7)
**PPV**	96.8%	(89.98–99.1)	75.7%	(59.9–86.64)	88.0%	(70.04–95.8)

### Prognostic value of FDG PET/CT, S100B and MIA in high risk melanoma patients

**Figure 1:**
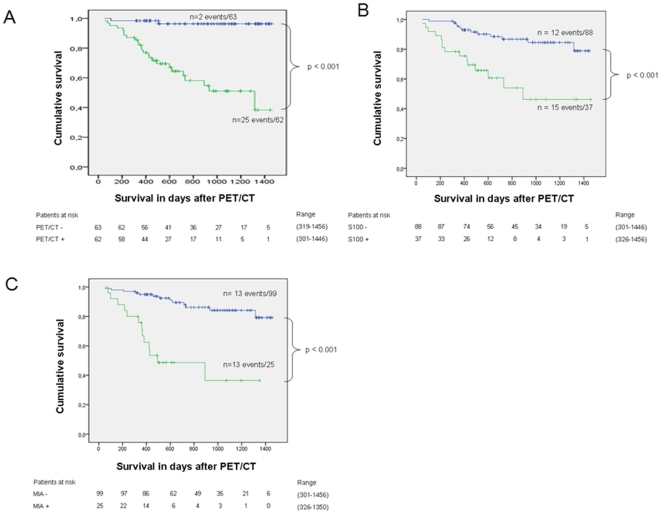
*Correlation of survival and positive findings* in PET/CT (A), S100 (B) MIA (C) and LDH (D). Blue: patients with positive findings. Green: patients diagnosed negative.

Kaplan-Meier survival curves are presented in Figure 1. In the group indicated as tumor-positive by PET 25 patients died (25 of 62 patients). In the group indicated as negative by PET 2 died (2 of 63 patients). By measuring S100B, 88 patients were denoted negative for recurrence and 37 positive. In the S100B-positive group 15 patients died (15 of 37 patients). In the S100B-negative group 12 patients died (12 of 88 patients). By measuring MIA, 99 patients were identified as negative for tumor and 25 patients were identified as positive. In the MIA-negative group 13 died from melanoma (13 of 99 patients). In the MIA-positive group 13 died (13 of 25 patients). The difference in melanoma associated mortality between patients with pathological findings in FDG PET/CT or elevated tumor markers and patients with normal tumor markers or non pathologic PET/CT was statistically significant (Cox regression, p<0.001). Patients with positive findings in PET/CT had a 17.2-fold higher mortality risk compared to patients with normal PET/CT. Patients with elevated MIA-, S100B- or LDH-levels had a 6.5-fold, 4.2-fold or 6.1-fold higher mortality risk compared to patients with normal tumor markers (Table 4). Analyses adjusted for age and gender revealed similar results (not shown). The median survival in the S100B-, MIA-, LDH-, or PET/CT-positive groups was 29.7 months (95% CI not determinable), 16.4 months (95% CI, 1.5 to 31.3), 16.4 months (6.7 to 26.1) or 43.87 months (19.6 to 68.1) (Table 4), indicating that in patients with truly positive tumor markers the survival is shorter, compared with patients who are positive in PET/CT only. The C index was highest for PET/CT indicating best discrimination (C-Index PET/CT: 0.93; LDH: 0.87; S100: 0.85; MIA: 0.88; Table 4). To test whether FDG-uptake in metastases is correlated to prognosis we determined the mean standardized uptake value (SUVmean) in all pathological lesions and correlated the SUV to the survival of patients. We found no statistically significant correlation of SUVmean and survival in our patient group taking M-stage and the bulk of disease into account (p = 0.158, HR = 0.943). We also analyzed if there is a quantitative relationship between S100- or MIA-level and survival. For S100 the Hazard ratio was 1.001 (95% CI 0.9997 to 1.0015, p = 0.189). Therefore, we did not find a statistically significant correlation of the S100 level and survival. For MIA the Hazard ratio was 1.046 (95% CI 1.026 to 1.066, p<0.001). These values indicate a statistically significant relation between the MIA-level and survival. In the group indicated as tumour positive, 45 patients received chemotherapy, 15 exclusively chemotherapy. 45 patients were treated with surgery, 14 exclusively with surgery; 29 patients received surgery and chemotherapy.17 patients were treated with radiation which was combined with chemotherapy in 16 cases and with surgery in one case. One patient refused any treatment. With regard to the low number of patients in these subgroups a correlation of treatment modality and prognosis could not be performed.

**Table 4:** Mortality risk (Hazard ratios) in patients positive for PET/CT, S100B, LDH or MIA.

	PET/CT	S100B	LDH*	MIA
**Increased mortality**	17.2	4.2	6.1	6.5
**p-value**	<0.001	<0.001	= 0.001	<0.001
**95% CI**	4.197–1.948	1.94–9.04	2.1–17.6	2.95–14.3
**Median survival**	43.87±21.37	29.7	16.4	16.4
**C-Index**	0.933	0.825	0.875	0.881
**95% CI**	0.654–0.990	0.531–0.951	0.464–0.983	0.581–0.975

*LDH was determined in a sub group of 58 patients.

### Correlation of the number of PET/CT-positive metastases and survival

We compared the melanoma associated mortality risk of patients with different numbers of metastases. Group-1 had no metastases, group-2 one metastasis, group-3 1–5 metastases and group-4 had more than 5 metastases. Kaplan-Meier survival curves were plotted for all four groups. In group-1 (63 patients) two died. In group-2 (23 patients) 7 died. In group-3 (14 patients) 6 died. In group-4 (25 patients) 12 died. Mortality rates were correlated with the number of metastases (p<0.001).

### Temporal relation of PET/CT findings and tumor marker positivity

For the analysis of the temporal relation of FDG PET/CT and tumor marker positivity we analyzed a sub-group of 42 patients truly positive for metastastatic disease according to the standard of reference. The tumor markers became positive after 538 days in median (range 8–7371) after initial diagnosis and PET/CT became positive in median after 565 days (range 125–829). In 13/42 patients (31%) metastatic disease was detected by PET but the tumor markers never became positive. In 12/42 patients (29%) tumor markers and PET/CT were found to be elevated at the same time. In 6/42 patients (14%) the tumor markers became positive later than FDG PET/CT. In 11/42 (26%) of patients the tumor markers became positive earlier compared to PET/CT. Therefore in a relevant number of patients with metastatic disease positive in FDG PET/CT the tumor markers were never elevated.

## Discussion

We analyzed the predictive values, sensitivity and specificity of FDG PET/CT compared with the tumor markers S100B and MIA in the follow-up of high risk melanoma patients. All three biomarkers detect metastases of malignant melanoma with clinically valuable sensitivity and specificity. Among these, FDG PET/CT is by far the most effective modality. Sensitivity and specificity of PET/CT were 96.8% each. Only two false positive od two false negative studies were found in our collective of melanoma patients. Both false negative patients had brain metastases which could not be detected due to the high physiological glucose metabolism in the brain, indicating that cMRI should be performed to exclude brain metastases in high risk melanoma patients. One false positive patient had a politeal neurinoma. The other false positive patient had a chromic lymphatic leukemia, mimicking retroperitoneal lymph node meatstases. The high sensitivity of FDG PET/CT to detect melanoma metastases is most likely due to the uniquely high glycolytic activity of melanoma cells leading to intensive FDG-uptake promoting the detectability of the lesions. The high FDG-uptake in almost all melanomas is a likely explanation for our finding that SUVmean is not correlated to survival of patients in our study collective in a statistically significant manner as it seems to be a feature of all melanomas and not to be restricted to highly aggressive subtypes [14]. Therefore, quantification of SUV does not further contribute to assessment of the mortality risk and the probability of survival as observed in our study.

FDG PET/CT is more sensitive and more specific in detection of metastases in high risk melanoma patients compared to the tumor markers S100B and MIA. S100B-concentration in the serum is also modulated by factors independent of melanoma reducing specificity to 85% [16], [17]. MIA has a high specificity of approximately 95%, but its low sensitivity of 36% causes a high rate of false negative findings [18]. In the present study an ROC analysis was conducted to evaluate diagnostic properties of S100B and MIA and to derive thresholds for identification of patients with metastatic or recurrent disease. Cut-off values found in our data as well as sensitivities and specificities using dichotomized markers S100B and MIA are consistent with those previously reported in literature [16], [17].

The lower sensitivity of the tumor markers compared to FDG PET/CT is puzzling. Presumably, the intense glucose metabolism in melanoma cells promotes the detection of relatively small tumor nodules which may be too small to produce enough tumor marker protein to elevate the serum level significantly. It is surprising that in FDG PET/CT negative patients, no patient showed elevated serum level of MIA or S100B or melanoma associated death during follow up. Therefore the macroscopic disease as evidenced by PET/CT seems to drive the prognosis and not microscopic disease.

Although sensitivity and specificity of FDG PET/CT and S100B have been compared in several studies the differences of prognostic values of these modalities have not been analyzed [14], [18], [19]. It is widely accepted that S100B and MIA are of prognostic value in stratification of survival probability in high risk melanoma patients. We found an inverted relationship between the serum level of S100B and MIA and survival in our collective [7], [8]. Positive findings in FDG PET/CT were associated with a significantly increased risk of melanoma associated death which was 17.2-fold higher compared to patients with normal FDG PET/CT. In comparison, patients with elevated S100B or MIA experienced a risk of only 4.2-fold or 6.5-fold higher. On the other hand we found that in patients with truly positive tumor markers the median survival is shorter compared to patients positive for recurrence only in PET/CT, indicating that S100B and MIA detect subgroups of metastasis with poor prognosis (table 4). The high predictive value of FDG PET/CT to assess the probability of melanoma associated mortality may be useful to stratify patients into groups which may benefit from novel therapies. It is particularly noteworthy that FDG PET/CT discriminates two groups with different mortality rates. As high risk melanoma is associated with a mortality risk of up to 50%, it is remarkable that PET negative patients within this group have a much better prognosis. As the mortality in PET-positive patients is higher both groups may require different treatment for example with novel molecular therapeutics such as mTOR inhibitors. This study demonstrates the superior prognostic value of FDG PET/CT and its higher power to discriminate between patient groups with different mortality risk in high risk melanoma compared to the tumor markers S100B and MIA. Our data suggest that prospective trials comparing the prognostic value of PET/CT, S100 and MIA should be performed. We also propose that for these studies the recently developed MR/PET should be used potentially improving the performance of PET as the problem of false negative PET-studies in patients with brain metastases may be overcome by the use of combined MR and PET.
